# Bovine Peripheral Blood Mononuclear Cells Are More Sensitive to Deoxynivalenol Than Those Derived from Poultry and Swine

**DOI:** 10.3390/toxins10040152

**Published:** 2018-04-11

**Authors:** Barbara Novak, Eleni Vatzia, Alexandra Springler, Alix Pierron, Wilhelm Gerner, Nicole Reisinger, Sabine Hessenberger, Gerd Schatzmayr, Elisabeth Mayer

**Affiliations:** 1 BIOMIN Research Center, Technopark 1, 3430 Tulln, Austria; barbara.novak@biomin.net (B.N.); alexandra.springler@biomin.net (A.S.); nicole.reisinger@biomin.net (N.R.); sabine.hessenberger@biomin.net (S.H.); gerd.schatzmayr@biomin.net (G.S.); 2 Institute of Immunology, Department of Pathobiology, University of Veterinary Medicine Vienna, 1210 Vienna, Austria; eleni.vatzia@vetmeduni.ac.at (E.V.); alix.pierron@vetmeduni.ac.at (A.P.); wilhelm.gerner@vetmeduni.ac.at (W.G.)

**Keywords:** mycotoxin, DON, deoxynivalenol, DOM-1, deepoxy-deoxynivalenol, lymphocyte proliferation, in vitro, immune system

## Abstract

Deoxynivalenol (DON) is one of the most prevalent mycotoxins, contaminating cereals and cereal-derived products. Its derivative deepoxy-deoxynivalenol (DOM-1) is produced by certain bacteria, which either occur naturally or are supplemented in feed additive. DON-induced impairments in protein synthesis are particularly problematic for highly proliferating immune cells. This study provides the first comparison of the effects of DON and DOM-1 on the concanavalin A-induced proliferation of porcine, chicken, and bovine peripheral blood mononuclear cells (PBMCs). Therefore, isolated PBMCs were treated with DON (0.01–3.37 µM) and DOM-1 (1.39–357 µM) separately, and proliferation was measured using a bromodeoxyuridine (BrdU) assay. Although pigs are considered highly sensitive to DON, the present study revealed a substantially higher sensitivity of bovine (IC50 = 0.314 µM) PBMCs compared to chicken (IC50 = 0.691 µM) and porcine (IC50 = 0.693 µM) PBMCs. Analyses on the proliferation of bovine T-cell subsets showed that all major subsets, namely, CD4^+^, CD8β^+^, and γδ T cells, were affected to a similar extent. In contrast, DOM-1 did not affect bovine PBMCs, but reduced the proliferation of chicken and porcine PBMCs at the highest tested concentration (357 µM). Results confirm the necessity of feed additives containing DON-to-DOM-1-transforming bacteria and highlights species-specific differences in the DON sensitivity of immune cells.

## 1. Introduction

Mycotoxins are fungal secondary metabolites that can cause disease and ultimately death in both humans and animals. According to mycotoxin surveys, up to 72% of the world’s agricultural commodities are contaminated by mycotoxins [1]. The trichothecene deoxynivalenol (DON), mainly produced by *Fusarium graminearum* and *Fusarium culmorum*, is one of the most prevalent of these mycotoxins, contaminating cereal and cereal-derived products worldwide [1,2]. Other studies have reported that silage is also a source of *Fusarium* metabolites and contributes to the total intake of DON in farm animals [3].

Due to its ability to interact with the 60S ribosomal subunit, DON interferes with protein synthesis, thereby predominantly damaging quickly proliferating cells, such as those of the immune system. Thus, following its passage through the gastrointestinal tract, the immune system presents an important target for the mycotoxin, leading to alterations in immune functions, dysregulation of the immune response, and impairments in the host’s resistance to pathogens [4]. 

In particular, DON negatively affects the proliferation and function of lymphocytes, including B, T, and natural killer (NK) cells. Low concentrations of the mycotoxin impair the activity of NK cells, which play an important role in the immune surveillance against tumors and microbial infections [5,6]. Furthermore, DON can activate (1–30 nM) or suppress (100–600 nM) mitogen-induced proliferation of human and animal lymphocytes [7]. High DON doses (>10 µM) trigger apoptosis of B and T cells, resulting in immunosuppression, vulnerability to infection, reactivation of latent infections, and decreased vaccine efficiency [8,9].

Deepoxy-deoxynivalenol (DOM-1), a microbial biotransformation product of DON, occurs naturally and due to the use of certain feed additives that contain DON-to-DOM-1-converting bacteria (e.g., genus *nov*. (formerly *Eubacterium*) sp. *nov*. BBSH 797) [10]. While only a few studies have investigated the in vitro effects of DOM-1 on immune cells, the severity of the effects of DON is known to be largely species-specific, due to variations in metabolism, absorption, and elimination [11,12]. Accordingly, pigs are considered to be the most DON-susceptible species, mainly due to the low bacterial habitation in the digestive system prior to the small intestine, as well as their limited metabolic activity and cereal-rich diet [13]. Hence, the European Commission (EC) has established very stringent guidelines with regard to the maximum DON concentration in complementary and complete feeding stuffs intended for pigs (0.9 mg/kg) [14]. In contrast, poultry (chickens, hens, turkeys, and ducks) are regarded as far more tolerable to dietary DON, which may possibly be due to the protective character of the renal first pass effect, which exists in poultry, as well as the high bacterial load located both before (in the crop) and after (in the cecum) the small intestine [15,16]. According to the EC guidelines, 5 mg/kg DON is permitted in complementary and complete feeding stuffs intended for poultry [14]. The assumed low sensitivity of poultry might explain why mycotoxin-infected cereal batches are frequently diverted to poultry feeding. Thus, poultry are the most heavily exposed species to DON out of all animals [17]. Chronic low dietary DON doses (<5 mg/kg diet) cause alterations in the immune system, lower productivity, and increased susceptibility to infectious diseases, without, however, showing an impact on performance [18]. Ruminants are regarded as quite resistant to DON, due to the rapid conversion of the toxin to its metabolite DOM-1 by rumen microbes such as BBSH 797 [19]. This strain can transform the epoxide group of trichothecenes, which is essential for the toxicity of DON, into a diene [20]. According to the EC guidelines, DON concentrations in complementary and complete feeding stuffs for ruminants can be as high as 5 mg/kg, with the exception of calves below the age of 4 months, where the guidance level is set at 2 mg/kg [14]. Acidosis prevalently occurs in cattle after the intake of starches from readily fermented carbohydrates, a common problem in ruminant production [13]. In dairy cows, subacute ruminal acidosis is an increasing problem that is nearly inevitable [21]. These high-concentrate diets may enhance the risk of toxin transfer across the damaged rumen mucosa into the blood stream. In this case, intact DON and its metabolites could potentially enter the systemic circulation [22]. 

Thus, while the effects of DON on proliferating lymphocytes have been reported, this study, for the first time, investigates the different degrees of sensitivity of porcine, chicken, and bovine peripheral blood mononuclear cells (PBMCs) to DON and DOM-1. We thereby provide important information regarding the species-specific susceptibility to this mycotoxin and its metabolite. 

## 2. Results

### 2.1. Porcine PBMCs

**Figure 1:**
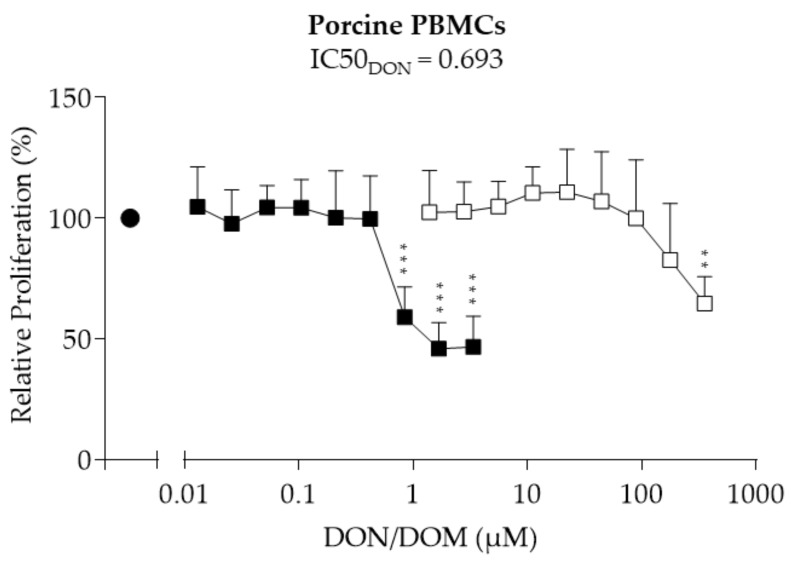
Relative proliferation (%) of porcine peripheral blood mononuclear cells (PBMCs) treated with deoxynivalenol (DON; ■) or deepoxy-deoxynivalenol (DOM-1; □). Freshly isolated porcine PBMCs were treated with DON (0.01–3.37 µM) or DOM-1 (1.39–357 µM) in the presence of concanavalin A (ConA; ● (1.25 µg/mL)) for 72 h. Proliferation was measured via bromodeoxyuridine (BrdU) proliferation assay and calculated relative to the ConA control, which was set as 100%. Data from experiments with PBMCs isolated from six different animals (mean + SD) are shown. Asterisks indicate significant differences compared to control (** *p* < 0.01, *** *p* < 0.001). IC50: half maximal inhibitory concentration.

The assessment of the effect of deoxynivalenol (DON) and its metabolite deepoxy-deoxynivalenol (DOM-1) on the proliferation of PBMCs was performed via bromodeoxyuridine (BrdU) assays (Figure 1). The proliferation of PBMCs was unaffected by DON at concentrations between 0.01 and 0.42 µM. A significant reduction in proliferation was observed at 0.84 µM (−41%, *p* = 0.012), 1.69 µM (−54%, *p* = 0.002), and 3.37 µM (−53%, *p* = 0.002) DON. A half maximal inhibitory concentration (IC50 value) of 0.693 µM was calculated for DON. DOM-1 did not negatively affect the proliferation of porcine PBMCs at concentrations between 1.39 and 178 µM. Only at 357 µM was proliferation reduced by 35% (*p* = 0.004).

### 2.2. Chicken PBMCs

**Figure 2:**
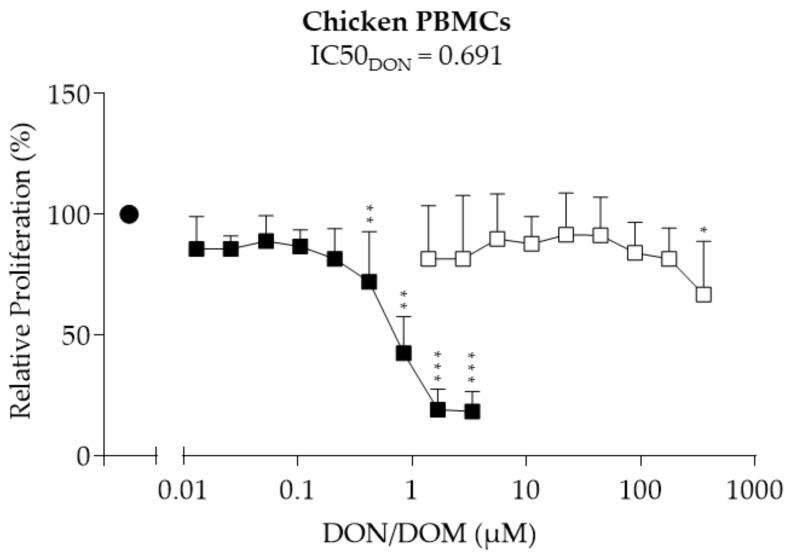
Relative proliferation (%) of chicken PBMCs treated with DON (■) or DOM-1 (□). Freshly isolated chicken PBMCs were treated with DON (0.01–3.37 µM) or DOM-1 (1.39–357 µM) in the presence of ConA (2.5 µg/mL) (●) for 28 h. Proliferation was measured via BrdU proliferation assays and calculated relative to the ConA control, which was set as 100%. Data from experiments with PBMCs isolated from eight (DON) and seven (DOM-1) different animals (mean + SD) are shown. Asterisks indicate significant differences compared to control (* *p* < 0.05, ** *p* < 0.01, *** *p* < 0.001).

We next assessed the effect of DON and its metabolite DOM-1 on the proliferation of chicken PBMCs (Figure 2). DON had no effect on proliferation at concentrations between 0.01 and 0.21 µM. A significant reduction in proliferation was observed between 0.42 (−28%, *p* = 0.007) and 3.37 µM (−83%, *p* = 0.000) DON. An IC50 of 0.691 µM was calculated for DON. While no effect was observed with 1.39 to 178 µM DOM-1, 357 µM significantly decreased the proliferation of chicken PBMCs by approximately 40% (*p* = 0.001).

### 2.3. Bovine PBMCs

**Figure 3:**
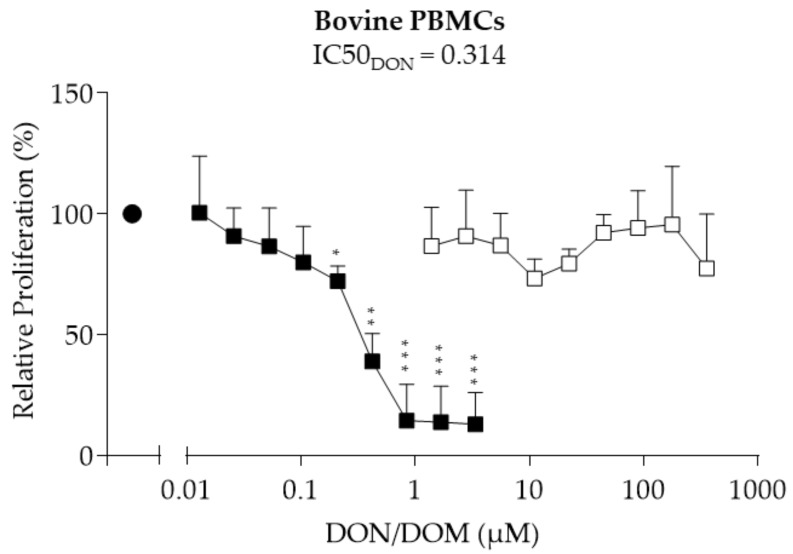
Relative proliferation (%) of bovine PBMCs treated with DON (■) or DOM-1 (□). Freshly isolated bovine PBMCs were treated with DON (0.01–3.37 µM) or DOM-1 (1.39–357 µM) in the presence of ConA (2.5 µg/mL) (●) for 72 h. Proliferation was measured via BrdU proliferation assays and calculated relative to the ConA control, which was set as 100%. Data from experiments with PBMCs isolated from six different animals (mean + SD) are shown. Asterisks indicate significant differences compared to control (* *p* < 0.05, ** *p* < 0.01, *** *p* < 0.001).

As a next step, the proliferation of bovine PBMCs treated with DON or DOM-1 was assessed. The exposure of bovine PBMCs to DON led to a significant dose-dependent decrease in proliferation at 0.21–3.37 µM (Figure 3). At 3.37 µM DON, proliferation was reduced by a maximum of 86% (*p* < 0.001). An IC50 value of 0.314 µM was calculated for DON. DOM-1 did not significantly influence the proliferation of bovine PBMCs at 1.39–357 µM.

### 2.4. Proliferation of Bovine T-Cell Subsets

**Figure 4:**
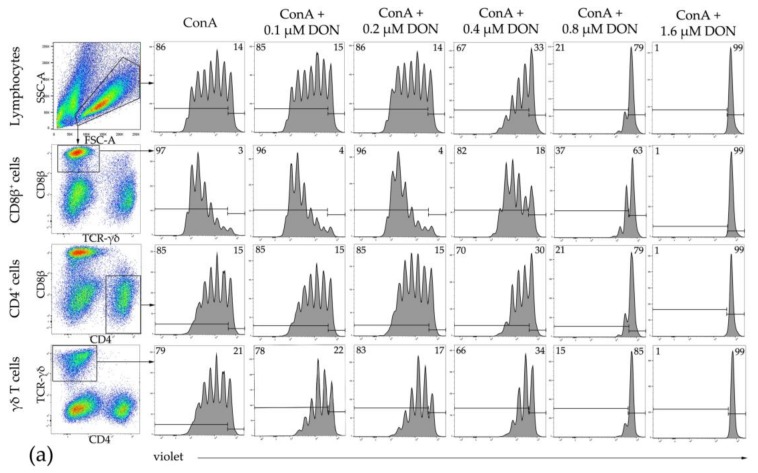

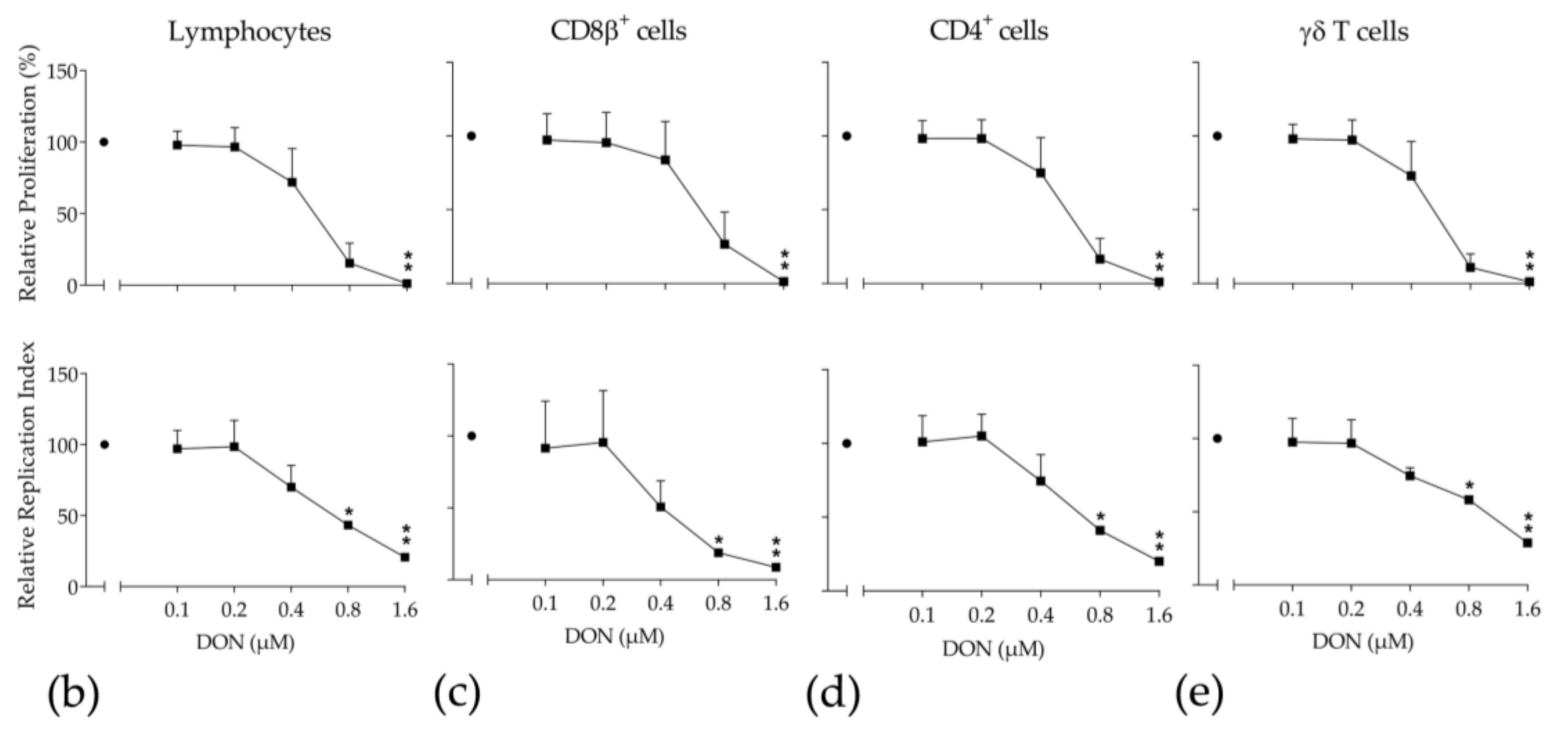
Proliferation of bovine lymphocytes and major T-cell subsets in the presence of various DON concentrations. PBMCs were stained with violet proliferation dye and cultivated in vitro in the presence of ConA and various DON concentrations for four days. After harvest, major T-cell subsets were labeled by antibodies against CD8β, CD4, and T-cell receptor (TCR)-γδ and analyzed by flow cytometry. (**a**) Lymphocytes were gated by light scatter properties and further subgated for the expression of CD8β, CD4, and TCR-γδ (pseudocolor plots). Histograms show the fluorescence intensities of the violet proliferation dye in lymphocytes (top) and the identified T-cell subsets under different cultivation conditions. Solid horizontal lines indicate the parental generation (on the right) and the proliferating generations (on the left). Numbers located in the two upper corners of the histograms indicate the frequency in % of the proliferating populations in comparison to the nonproliferating cells. Representative data from one animal out of six are shown. (**b**–**e**) Relative proliferation (top row) and relative replication index (bottom row) are shown for total lymphocytes (**b**), CD8β^+^ cells (**c**), CD4^+^ cells (**d**), and TCR-γδ^+^ T cells (**e**) for different DON concentrations (0.1, 0.2, 0.4, 0.8, and 1.6 µM) (■) in comparison to ConA alone (●). Proliferation and replication index values obtained in ConA cultures were set as 100%. Data from experiments with PBMCs isolated from six different animals (mean + SD) are shown. Asterisks indicate significant differences compared to control (* *p* < 0.05, ** *p* < 0.01). SSC-A side scatter area, FSC-A: forward scatter area.

Having identified the lowest IC50 value for DON in bovine PBMCs, we next investigated whether the predominant T-cell subsets in cattle are particularly sensitive to this mycotoxin. Hence, bovine PBMCs were labeled with a proliferation dye, and major T-cell subsets were identified by surface staining of CD4, CD8β, and T-cell receptor (TCR) γδ (Figure 4). In these experiments, PBMCs were cultivated either in the presence of concanavalin A (ConA) alone or in combination with different DON concentrations (0.1, 0.2, 0.4, 0.8, and 1.6 µM). Lymphocyte blast cells were identified by light scatter properties and analyzed for proliferation (Figure 4a, top row), and further subgated for the expression of CD8β, CD4, and TCR-γδ. These subpopulations were again analyzed for proliferation (Figure 4a: 2nd, 3rd, and 4th row). The percentage of proliferating cells was identified by gating (Figure 4a: histograms; horizontal bars to the left represent proliferating cells), whereas the replication index, indicating the fold expansion of responding cells over time, was calculated by the proliferation modeling tool of FlowJo software. The number of proliferating cells and the replication index of ConA treated samples were set as 100%, and the relative proliferation and replication index values were calculated for cultures cotreated with the various DON concentrations. Overall, lymphocytes and each T-cell subset showed similar reductions in proliferation and replication index values (Figure 4b–e). The first reduction in proliferating cells and replication index values was observed at 0.4 µM DON, although these reductions did not reach significance. Reductions were more pronounced at 0.8 µM of DON, but reached significance only for the replication index. At 1.6 µM of DON, proliferation was almost completely abolished, resulting in a strong reduction of the relative proliferation and replication index values. The IC50_DON_ values of the proliferating cells were 0.52 µM for total lymphocytes, 0.64 µM for CD8β^+^ cells, 0.53 µM for CD4^+^ cells, and 0.50 µM for γδ T cells, again indicating that all investigated T-cell subsets were affected to a similar extent.

## 3. Discussion

The present in vitro study clearly demonstrates that porcine, chicken, and bovine PBMCs possess different sensitivities to deoxynivalenol (DON). Although cattle are considered particularly resistant to mycotoxins, bovine PBMCs were far more sensitive to the inhibitory effects of DON on their proliferation than chicken or porcine PBMCs. Furthermore, in agreement with several other studies [23,24,25,26,27,28], the toxic effect of DON was strongly attenuated through de-epoxidation to its metabolite deepoxy-deoxynivalenol (DOM-1). 

We examined the in vitro effects of DON and DOM-1 on the proliferation of ConA-stimulated porcine, chicken, and bovine PBMCs. Plant-derived mitogens such as ConA, phytohemagglutinin (PHA), and pokeweed mitogen act differently on lymphocytes; for example, ConA and PHA stimulate T cells, while others, such as pokeweed, activate B and T cells [29,30]. We recorded changes in the proliferative capacity of cells by measuring DNA synthesis via the incorporation of the thymidine analog bromodeoxyuridine (BrdU) into newly synthesized DNA. PBMC proliferation is frequently quantified via the convenient MTT and lactate dehydrogenase (LDH) assays, though these provide nonspecific measurements of cell proliferation. The MTT response, for example, may vary considerably in viable cells depending on their metabolic state [31]. As demonstrated by Goyarts et al. [32] and Charoenpornsook et al. [33], DNA synthesis-based methods, such as BrdU or [^3^H]-thymidine incorporation, provide a far more sensitive measurement of lymphocyte proliferation than LDH, MTT, or trypan blue exclusion methods.

Interestingly, the measurement of the highly sensitive BrdU incorporation showed that among porcine, chicken, and bovine PBMCs, bovine cells were the most sensitive to DON in terms of the inhibition of lymphocyte proliferation. DON significantly decreased bovine lymphocyte proliferation at 0.21 µM with an IC50 of 0.314 µM. In comparison, the proliferation of chicken- and porcine-derived PBMCs was significantly reduced at 0.42 µM (IC50 = 0.691 µM) and 0.84 µM (IC50 = 0.693 µM), respectively. These findings are in agreement with Charoenpornsook et al. [33], who reported a 50% inhibition of bovine PBMC proliferation at 0.24 µM, using the [^3^H]-thymidine incorporation method. Dänicke et al. [22] investigated the proliferation of bovine PBMCs in response to DON via the MTT assay and reported an IC50 of 0.5 µM DON. According to the authors, the age of the cows had an influence on their susceptibility: PBMCs isolated from calves were more sensitive than those isolated from adult cows. Similarly, Wada et al. [34] reported a 25% proliferation reduction in PHA-stimulated adult cow PBMCs, compared to a 55% reduction in calf PBMCs, following treatment with 3.37 µM DON for 72 h. Therefore, in our study, PBMCs of fetal and young animals were excluded to avoid possible effects of age-derived sensitivities.

The proliferation dye carboxyfluorescein diacetate succinimidyl ester (CFDA-SE) and its derivatives such as the violet proliferation dye allow a simultaneous analysis of proliferation and cell phenotype [35], a method that is also well established for bovine T cells [36]. We made use of this approach to investigate potential differences in DON sensitivity between CD4^+^, CD8β^+^, and γδ T cells in bovine-derived PBMCs. The results indicated that all three major T-cell subsets showed a similar impairment in proliferation when stimulated by ConA. These findings suggest that the inhibitory effect of DON on ConA-induced T-cell proliferation in all three T-cell subsets is based on the same mechanism.

The IC50_DON_ values obtained for bovine total lymphocytes and the derived T-cell subsets were somewhat higher than those obtained in BrdU assays (0.314 µM for BrdU and 0.5 to 0.6 µM for total lymphocytes and T-cell subsets). This discrepancy is probably attributable to the different methodologies: BrdU analysis addresses DNA synthesis, whereas proliferation dyes assess a loss of fluorescence intensity, resulting from the development of daughter cells that bear a reduced number of proliferation dye-labeled proteins in their cytoplasm. Notably, the IC50 values obtained in the violet proliferation assays for bovine PBMCs were still below the BrdU IC50 values for chicken and porcine lymphocytes.

The high DON sensitivity of bovine PBMCs is relevant [37] with respect to several aspects that are often insufficiently considered. It is frequently suggested that ruminants are resistant to mycotoxins due to the composition of their intestinal microbiota, which is capable of converting DON to its metabolite DOM-1 before it reaches the bloodstream. However, it is often left unconsidered that some mycotoxins found in silage and stored feedstuffs possess antimicrobial characteristics, through which they can modify the ruminal microbiota, thereby reducing its detoxification capacity. Consequently, contaminated digesta can reach the duodenum, where contaminants are absorbed into the bloodstream [38]. Moreover, high-concentrate diets, frequently used in high-performance cows, enhance the development of rumen acidosis. The latter can damage the barrier function of the rumen wall [13,39], a phenomenon associated with the translocation of rumen bacteria (e.g., *Fusobacterium necrophorum*) to the liver via the portal vein as well as the transport of harmful substances into the bloodstream [22,40,41]. Furthermore, He et al. [42] demonstrated that DON transformation is inhibited by pH values below 5.2 due to the inactivation of microorganisms in acidic conditions or by specific inhibitory effects on the de-epoxidation process. Consequently, not only DOM-1, which we report to have no effect on bovine PBMCs up to a concentration of 357 µM, but also intact DON as well as its acetylated derivatives may enter systemic circulation. The entrance of DON into the bloodstream can trigger nonspecific clinical symptoms such as metabolic and hormonal imbalances, inflammation, and immune responses. In serum, maximal values of 0.03 µM DON [22] and 0.06 µM DON [43] were found in cattle fed DON-contaminated feed. Interestingly, in one study assessing the serum of dairy cows, concentrations up to ~0.2 µM DON were found along with an increased incidence of mastitis [44]; thus reflecting our in vitro values. In fact, due to a wide range of mycotoxin-producing fungi, their varied distributions in feed, the multidirectional effects on the host, and the high cost of laboratory analysis, mycotoxicosis is difficult to diagnose. Moreover, the detection of feed contaminated with mycotoxins is challenging, and mycotoxicosis may be suspected in all disease cases that display nonspecific symptoms and high resistance to conventional treatment [45].

Although less susceptible when compared to bovine PBMCs, the proliferative capacity of both porcine and chicken lymphocytes was strongly affected by DON. Despite several studies characterizing the effect of trichothecenes such as DON on immune parameters, we are the first to show the effect of DON and DOM-1 on chicken lymphocyte proliferation in vitro. Generally, the high bacterial content in poultry, which converts toxic DON to nontoxic DOM-1 before the small intestine, strongly decreases the amount of DON that reaches the small intestine, making the animal more robust against oral intoxication [4,16]. Reports additionally suggest that in poultry, only a small amount of ingested DON (~19%) actually reaches the small intestine as a native toxin [46]. It was recently also reported that DON-3α-sulfate is a major detoxification metabolite in chickens, accounting for approximately 88.6% of the administered doses of DON. Accordingly, the ability of chickens to biotransform DON to DON-sulfates could explain the reduced DON susceptibility of poultry [47]. Nevertheless, in accordance with our findings, literature reports indicate that DON impairs immune function in poultry. Exposure to a DON-contaminated diet, for example, reduced serum antibody titers against Newcastle disease virus in laying hens and broilers [48,49]. Diets containing high levels of *Fusarium*-toxin-contaminated grain fed to broilers have been shown to decrease the peripheral blood monocyte and B cell counts without altering serum immunoglobulin concentrations [50]. Other studies suggest that *Fusarium* mycotoxins in feed can decrease the level of blood leukocytes, B lymphocytes, and CD4^+^ and CD8^+^ T cells [51]. In a broiler study, Ghareeb et al. [52] reported a decrease in the plasma levels of tumor necrosis factor α as well as a downregulation of interleukin 1β, transforming growth factor β receptor 1, and interferon-γ gene expression in response to DON. The authors concluded that DON provokes and modulates immunological reactions in broilers, which could increase disease susceptibility.

With respect to the DON-induced inhibition of porcine PBMC proliferation, our findings confirm those of Taranu et al. [7], who reported that while DON exerted stimulating effects on porcine PBMC proliferation at concentrations up to 0.33 µM, further increases in the concentrations of the mycotoxin dose-dependently reduced proliferation. The authors reported considerable inhibition of cell proliferation at concentrations between 3.37 and 33.7 µM DON. Furthermore, Goyarts et al. [53] found significant reductions in porcine PBMC proliferation using 0.94 and 1.89 µM DON, with IC50 values of 1.04 µM (MTT assay) and 0.67 µM (BrdU assay). According to a study by Dänicke et al. [27], even 0.7 µM DON significantly reduced the viability of ConA-stimulated porcine PBMCs. Maximum proliferation decreases of approximately 80% were observed at 2.7 µM. In accordance with our findings, the same authors reported DOM-1 to have no effect on porcine proliferation up to a concentration of 22.9 µM.

## 4. Conclusions

In our study, DON inhibited lymphocyte proliferation in all three tested species, where the greatest decrease was seen in bovine PBMCs, followed by chicken and porcine PBMCs. This decrease can most likely be attributed to the capacity of the mycotoxin to cause ribotoxic stress and inhibit protein synthesis. We herein hypothesize that DON also might pose a risk factor to the immune system of animals that are frequently considered to possess a low DON susceptibility, such as ruminants and poultry. This effect could indicate that under certain conditions, for instance inflammation or gastric diseases, low dietary DON concentrations might impair animal health by acting as an immunosuppressant.

## 5. Materials and Methods 

### 5.1. Cell Isolation

To isolate peripheral blood mononuclear cells (PBMCs), fresh porcine, chicken, and bovine whole blood was obtained from an abattoir in EDTA-containing (120 mg/mL) (Sigma Aldrich, St. Louis, MO, USA) centrifuge tubes and diluted 1:2 with phosphate buffered saline (PBS) (Gibco, Life Technologies, Carlsbad, CA, USA). Information on sex, breed, and history was not available. Pigs were slaughtered at an age of approximately 6.5 months, chicken at an average age of 70 days, and cattle at an age of approximately >18 months. Exact age from individual animals is unknown, but all used study animals had reached at least late adolescence. Of this mixture, 35 mL was gently layered onto 15 mL Ficoll-Paque^TM^ Plus (GE Healthcare, Little Chalfont, UK) in 50 mL centrifuge tubes and centrifuged at 300× *g* without brake for 30 min. PBMCs, located in the middle of the obtained layers, were carefully aspirated and transferred into a fresh 50 mL centrifuge tube. Cells were washed three times with 40 mL PBS and cell numbers were determined via Tuerk solution (Sigma Aldrich, St. Louis, MO, USA) staining. Cells were used for assays immediately after isolation (no prior cryopreservation).

### 5.2. Deoxynivalenol (DON) and Deepoxy-Deoxynivalenol (DOM-1) Treatment

Approximately 1 × 10^5^ porcine PBMCs or 4 × 10^5^ chicken and bovine PBMCs were seeded in RPMI-1640 culture medium supplemented with 25 mM 4-(2-hydroxyethyl)-1-piperazineethanesulfonic acid) (HEPES), 100 U/mL penicillin, 100 µg/mL streptomycin, 0.25 µg/mL amphotericin B (all Sigma Aldrich, St. Louis, MO, USA), 2 mM L-glutamine, and 5% heat-inactivated fetal bovine serum (FBS) (both Gibco, Life Technologies, Carlsbad, CA, USA) in round-bottom 96-well microplates (Eppendorf, Hamburg, Germany). DON (Biopure, Romer Labs^®^, Tulln, Austria) was dissolved in distilled water to a concentration of 6.75 mM and DOM-1 (Biopure, Romer Labs^®^, Tulln, Austria) was obtained in acetonitrile at a concentration of 180.1 µM. DOM-1 was first evaporated with nitrogen and DON and DOM-1 were further diluted with respective media to achieve the desired concentrations of 0.01–3.37 µM for DON and 1.39–357 µM for DOM-1. The calculated solubility (Marvin Software Version 17.9.0, 2017, ChemAxon (http://www.chemaxon.com)) in water at pH 7 was ~49 mM for DON and ~40 mM for DOM-1; thus, the concentrations used were far below the solubility threshold. Furthermore, the stability of DON and DOM-1 were confirmed in medium in cell culture experiments [54].

Cells were immediately treated with DON (0.01–3.37 µM) or DOM-1 (1.39–357 µM) in the presence of 1.25 µg/mL (porcine PBMCs) or 2.5 µg/mL (chicken and bovine PBMCs) ConA (Sigma-Aldrich, St. Louis, MO, USA). Cells were incubated at 39 °C for 28 h (chicken PBMCs) or at 37 °C for 72 h (porcine and bovine PBMCs). The optimal seeding density, incubation temperature, and incubation time have been assessed in pilot experiments to assure the highest proliferation rates (data not shown). Control cells were treated with fully supplemented culture medium with (positive control) and without (negative control) ConA at each concentration.

### 5.3. Cell Proliferation ELISA, BrdU Assay

Relative proliferation was determined via the cell proliferation ELISA, BrdU assay (Roche, Rotkreuz, Switzerland). The assay was performed according to the manufacturer’s instructions. Briefly, cells were labeled with BrdU labeling solution for 4 h, and the microplate was subsequently centrifuged at 300× *g* for 10 min. The labeling medium was removed, and cells were completely dried and fixed with the provided fixing solution for 30 min at room temperature. Subsequently, anti-BrdU-peroxidase solution was added to each well for 90 min at room temperature. Then, the antibody conjugates were removed and cells were washed three times with the provided washing solution. After treatment with the substrate solution, the reaction was stopped with 1 M sulfuric acid, and optical density (OD) was measured at 450 nm and 690 nm (reference wavelength) by a microplate reader (Biotek Instruments, Inc., Winooski, VT, USA). The highest measured OD values for ConA-stimulated PBMCs were around ~1.1 (mostly, OD values were between 0.6–0.9). For the cell control (PBMCs only), OD values were around 0.04–0.09. The proliferation response is expressed as the stimulation index (SI), calculated as described in Equation 1. According to literature, a stimulation index of >2 was considered as positive [55]. Therefore, experiments where ConA-stimulated PBMCs did not fulfill this performance standard were excluded.

(1) $$\mathrm{Stimulation}\;\mathrm{index}\;\left(\mathrm{SI}\right)=\frac{\mathrm{OD}\;\left(\mathrm{mean}\;\mathrm{of}\;\mathrm{treated}\;\mathrm{cells}\right)}{\mathrm{OD}\;\left(\mathrm{mean}\;\mathrm{of}\;\mathrm{negative}\;\mathrm{control}\right)}$$

Equation (1): Calculation of the stimulation index (SI).

To exclude individual variations between animals, relative proliferation was calculated as follows (Equation (2)):

(2) $$\mathrm{Relative}\;\mathrm{Proliferation}\;\left(\%\right)=\frac{\left(\mathrm{SI}\;\left(\mathrm{treated}\;\mathrm{cells}\right)\right)}{\left(\mathrm{SI}\;\left(\mathrm{ConA}\right)\right)}*100$$

Equation (2): Calculation of the relative proliferation (%).

### 5.4. Cell Proliferation and Phenotyping of Bovine PBMCs by Violet Proliferation Assays

The isolation protocol for bovine lymphocytes used in the violet proliferation assays was the same as described above, with the exception that the centrifugation on the Ficoll-Paque gradient was performed at 920× *g* for 30 min. Following isolation, PBMCs used in violet proliferation assays underwent a freezing/thawing procedure as described by Leitner et al. [56].

Following thawing, bovine PBMCs were washed in PBS and counted in trypan blue. Cell viability was between 77 and 90%. The PBMCs were labeled with a CellTrace^TM^ Violet Cell Proliferation Kit (Thermo Fisher Scientific, Waltham, MA, USA) as follows. The cell number was adjusted to 2 × 10^7^ in 1 mL of PBS, and 1 mL of violet dye solution was added to the cells (resulting in a violet dye concentration of 5 µM), followed by incubation at 37 °C for 10 min in a water bath. To stop the violet dye uptake, 2 mL of FBS (PAN-Biotech, Aidenbach, Germany) was added and incubation continued for 15 min at room temperature in the dark. Finally, cells were washed three times in culture medium (RPMI 1640 + 10% heat-inactivated FBS + 100 U/mL penicillin, 100 µg/mL streptomycin, all from PAN-Biotech), followed by counting and plating at 2 × 10^5^ cells per well. Cells were cultivated in the presence of ConA (3 µg/mL, Amersham Biosciences, Uppsala, Sweden) with or without different DON concentrations (0.1, 0.2, 0.4, 0.8, and 1.6 µM) in round-bottom 96-well plates at 37 °C for four days. 

After the in vitro cultivation, cells were analyzed for proliferation and surface marker expression by four-color staining flow cytometry. The following primary antibodies were used: anti-CD8β (clone CC58, VMRD, Pullman, WA, USA), anti-CD4 (clone CC8, AbD Serotec, Kidlington, UK), and anti-TCR-γδ (clone GB21A, VMRD). These primary antibodies were labeled by the following isotype-specific secondary antibodies: goat anti-mouse IgG1 Alexa647 (Thermo Fisher Scientific, Waltham, MA, USA), goat anti-mouse IgG2a-PE (Southern Biotech, Birmingham, AL, USA), and goat anti-mouse IgG2b-Alexa488 (Thermo Fisher Scientific, Waltham, MA, USA), respectively. Initially, cells were harvested and washed once in PBS + 3% FBS. Cells were stained with primary and secondary antibodies in two consecutive incubations performed at 4 °C for 20 min in 96-well round-bottom plates. Between the first and the second incubation and after the second incubation, cells were washed twice in PBS + 3% FBS. Cells were finally resuspended in the same buffer and analyzed by a FACSCanto II flow cytometer (BD Biosciences, San Jose, CA, USA). Between 5 × 10^4^ and 2 × 10^5^ PBMCs were acquired per sample. Flow cytometry data were analyzed and processed by FlowJo software version 10.3 (FlowJo LLC, Ashland, OR, USA).

### 5.5. Statistics

Statistical analysis was performed with IBM^®^ SPSS (Version 19.0, IBM Corp., New York, NY, USA, 2010). Values from independent experiments are expressed as the means of 6–8 replicates ± standard deviation (SD). One replicate means isolating and testing the PBMCs of one individual animal in triplicate. The stimulation index and the relative values were calculated and the relative means of the replicates ± SD were plotted and used for statistical evaluation. All values were analyzed for normality (Shapiro–Wilk) and homogeneity of variance (Levene statistics). Normally distributed homogenous data were analyzed by analysis of variance (ANOVA) and Dunnett’s *t*-tests and compared to control data. If data did not follow a normal distribution, a Kruskall–Wallis test was used. IC50 values were calculated with relative numbers using GraphPad Prism (Version 7.0, GraphPad Software, Inc., La Jolla, CA, USA, 2017). In short, data was normalized and then a four-parameter nonlinear regression curve (log (inhibition) versus response with variable slope (least squares ordinary fit, with the condition that the Hillslope is <0) was applied to calculate the IC50 values.
